# Naloxone Availability and Cost After Transition to an Over-the-Counter Product

**DOI:** 10.1001/jamahealthforum.2024.1920

**Published:** 2024-07-26

**Authors:** Grace T. Marley, Izabela E. Annis, Kathleen L. Egan, Paul Delamater, Delesha M. Carpenter

**Affiliations:** 1 UNC Eshelman School of Pharmacy, Chapel Hill, North Carolina; 2 South East Area Health Education Center, Wilmington, North Carolina; 3 Division of Pharmaceutical Outcomes and Policy, Eshelman School of Pharmacy, University of North Carolina at Chapel Hill; 4 Department of Implementation Science, Division of Public Health Sciences, Wake Forest University School of Medicine, Winston-Salem, North Carolina; 5 Department of Geography and Environment, University of North Carolina at Chapel Hill; 6 UNC Eshelman School of Pharmacy, Asheville, North Carolina

## Abstract

**Importance:**

The US Food and Drug Administration approved Narcan, a nasal spray formulation of naloxone, for sale as an over-the-counter (OTC) medication in March 2023. The purpose of OTC approval was to improve naloxone accessibility to reduce opioid overdoses; however, research has not yet evaluated whether naloxone’s availability and cost changed since this policy was implemented.

**Objective:**

To evaluate whether the accessibility and cost of naloxone at North Carolina community pharmacies changed after OTC naloxone became available and whether cost and availability varied by pharmacy type and urbanicity.

**Design, Setting, and Participants:**

This longitudinal telephone-based secret shopper survey study included a stratified sample of 202 North Carolina community pharmacies, including health department, independent, and chain pharmacies. There were 2 separate data collection efforts from March to April 2023 (before OTC naloxone could be sold at pharmacies) and November 2023 to January 2024 (after OTC naloxone was sold at pharmacies).

**Exposure:**

OTC naloxone first became available for sale at community pharmacies in September 2023.

**Main Outcomes and Measures:**

The main outcomes were same-day availability of naloxone without a clinician-issued prescription and the quoted out-of-pocket cost for cash-paying patients.

**Results:**

Data were collected from 192 pharmacies. Same-day naloxone availability increased from 42.2% (81 of 192) before OTC naloxone availability to 57.8% (111 of 192) after (*P* < .001). The mean (SD) quoted out-of-pocket cost decreased from $90.93 ($42.6) pre-OTC availability to $62.67 ($41.0) post-OTC availability (*P* < .001). Independent pharmacies had higher mean (SD) costs than chain pharmacies in both the pre-OTC phase ($109.47 [$37.90] vs $86.40 [$35.70]; *P* < .001) and post-OTC phase ($77.59 [$38.90] vs $57.74 [$35.90]; *P* = .004). Out-of-pocket costs did not differ by urbanicity in the pre-OTC phase; however, mean (SD) costs were higher at suburban ($88.67 [$66.80]) and rural ($65.43 [$35.00]) pharmacies compared with urban pharmacies ($53.58 [$29.00]) in the post-OTC phase (*P* = .003).

**Conclusions and Relevance:**

The Food and Drug Administration’s approval of OTC naloxone nasal spray contributed to an increase in pharmacy-based availability of naloxone and a reduction of its cost for cash-paying patients. Cost was higher at independent pharmacies compared with chain pharmacies and lower in urban pharmacies compared with suburban and rural pharmacies.

## Key Points

**Question:**

How did pharmacy-based access to naloxone and quoted out-of-pocket cost for naloxone change after naloxone became available as an over-the-counter (OTC) product?

**Findings:**

In this secret shopper survey study of 192 North Carolina pharmacies, same-day availability of naloxone without a clinician-issued prescription increased from 42.2% of pharmacies before OTC availability to 57.8% after. The mean cost of naloxone decreased from $90.93 to $62.67 after 1 formulation of naloxone became available OTC.

**Meaning:**

These findings suggest that the introduction of OTC naloxone increased naloxone availability and decreased quoted out-of-pocket cost for cash-paying patients at pharmacies in North Carolina.

## Teaser

This secret shopper survey study evaluates whether the accessibility and cost of naloxone at North Carolina community pharmacies changed after over-the-counter naloxone became available and whether cost and availability varied by pharmacy type and urbanicity.

## Introduction

Opioid overdose is a major public health concern in the US, resulting in nearly 83 000 drug overdose deaths in the 12 months ending in December 2022.^1^ Naloxone is a lifesaving medication that acts quickly to reverse opioid overdoses and restore breathing. Prior to March 2023, naloxone was a prescription-only product; however, to increase access to naloxone, all 50 US states and the District of Columbia have passed laws that allow individuals to obtain naloxone at pharmacies by means other than a clinician-issued prescription.^2^ For example, North Carolina implemented a statewide standing order in 2016 that authorized pharmacists to dispense naloxone to patients without an individual clinician-issued prescription.^3^

Despite policies to increase naloxone access at pharmacies, its availability has remained suboptimal.^4,5,6,7,8,9,10,11,12,13,14,15^ Secret shopper studies, studies in which individuals pose as patients to evaluate access to care, found that same-day access to naloxone varied from as low as 23.5% of pharmacies in California to as high as 96.1% of pharmacies in Massachusetts.^4,5,6,7,8,9,10,11,12,13^ When comparing naloxone access by pharmacy type, independent pharmacies, or pharmacies that follow the small owner/operator model, have lower naloxone availability compared with chain pharmacies, or pharmacies that are part of a corporation or have more than 5 locations.^4,6,9,15,16,17,18,19,20^ Urban pharmacies demonstrated higher accessibility to naloxone compared with rural pharmacies.^8^ Pharmacy-based access to naloxone is important in rural areas because harm reduction organizations that distribute naloxone for free are often less accessible.^21^ Reasons for limited pharmacy-based naloxone access include inadequate stocking of naloxone, stigma surrounding addiction and overdose, lack of patient demand, lack of naloxone training, and high cost.^4,16,17,18,19,22^

Even in pharmacies that had naloxone available by standing order, cost remained a significant barrier, especially for uninsured individuals. Data from the 2022 National Survey on Drug Use and Health indicate that 11.5% of individuals with substance use disorder were uninsured, which is significant when the mean cost of naloxone increased 606% among uninsured individuals from 2010 to 2018.^23,24^ Secret shopper studies have documented high out-of-pocket costs for naloxone nasal spray, averaging more than $125, with elevated out-of-pocket costs at independent pharmacies compared with chain pharmacies and in rural areas compared with urban areas.^4,5,9,10,15,23,24,25,26^

North Carolina has a high opioid overdose death rate, particularly in rural areas.^1,27^ In 2019, a secret shopper study of North Carolina pharmacies found that 61.7% were willing to dispense naloxone without a prescription, in accordance with the statewide standing order. The study also found that the mean quoted out-of-pocket cost for naloxone nasal spray was $123.24.^12^ Moreover, the results of the first phase of this study conducted in 2023 found that only 53% of pharmacies were willing to dispense naloxone without a prescription, with an average cost of $96.27.^12,15^

In an effort to increase the public’s access to naloxone, the US Food and Drug Administration approved Narcan (Emergent BioSolutions), a nasal spray formulation of naloxone, for sale as an over-the-counter (OTC) product in March 2023, with the product first becoming available at pharmacies in September 2023.^28^ The suggested manufacturer’s retail price for 2 doses of OTC naloxone nasal spray, 4 mg, was $45.99.^29^ This price is significantly lower than the $125 mean price of naloxone documented previously, suggesting the average price of naloxone may be lower.^4,5,9,10,15,25,26^ However, to our knowledge, no studies to date have examined whether the quoted out-of-pocket cost of naloxone has decreased since the OTC product became available. Additionally, no research has documented naloxone accessibility at pharmacies since the OTC product became available. This longitudinal secret shopper survey study aimed to document whether naloxone access and cost at North Carolina pharmacies changed after naloxone became available for OTC sale. We hypothesized that naloxone would be more accessible and less costly after the introduction of OTC naloxone. We also hypothesized that naloxone would have greater same-day accessibility at chain and urban pharmacies.

## Methods

### Study Design and Setting

We used a longitudinal telephone-based secret shopper survey study design. The secret shopper methodology was chosen due to its ability to study health care access from a realistic patient-centered perspective. Data were collected in 2 phases, with the first phase being from March to April 2023 (before OTC naloxone availability) and the second phase being conducted from November 2023 to January 2024 (after OTC naloxone availability).^15^ The protocol was deemed exempt under federal regulations as non-human subjects research by the University of North Carolina at Chapel Hill Institutional Review Board.

### Study Sample

To make our results as comparable with a previous 2019 North Carolina naloxone secret shopper study as possible, we attempted to sample the same 200 pharmacies that were included in that study.^12^ The 23 pharmacies that had closed since 2019 were replaced with randomly selected pharmacies stratified by pharmacy type to have an equal distribution of independent and chain pharmacies.^12^ Additionally, the sample of 202 pharmacies was stratified to include at least 1 pharmacy from each North Carolina county. The sampling frame for newly selected pharmacies was derived from a list of all active chain (n = 1206), independent (n = 638), and health department (n = 8) pharmacies, as documented by the North Carolina Board of Pharmacy (NCBOP). This list was cross-referenced with the October 2022 Hayes list of retail pharmacies in North Carolina to create a comprehensive list of community pharmacies.^30^ Pharmacy type was determined using the designation from the NCBOP list.^15^ Like the 2019 secret shopper study,^12^ all North Carolina health departments with a physical pharmacy and pharmacy phone number were included in the sample because community members can access naloxone at those pharmacies in the same way they can access naloxone from chain and independent community pharmacies.

### Secret Shopper Training and Data Collection

Data were collected in 2 phases. The first phase was conducted from March to April 2023 by a team of 6 trained secret shoppers (2 volunteer students and 4 paid research assistants, including 1 of us [G.T.M.]). During the second phase of the study, data were collected by a team of 9 trained shoppers (1 postdoctoral research associate [G.T.M.], 4 volunteer students, and 4 paid research assistants) from November 2023 to January 2024. Although OTC approval was granted in March 2023, OTC naloxone was not available for purchase by pharmacies until September 2023.

**Figure 1.**
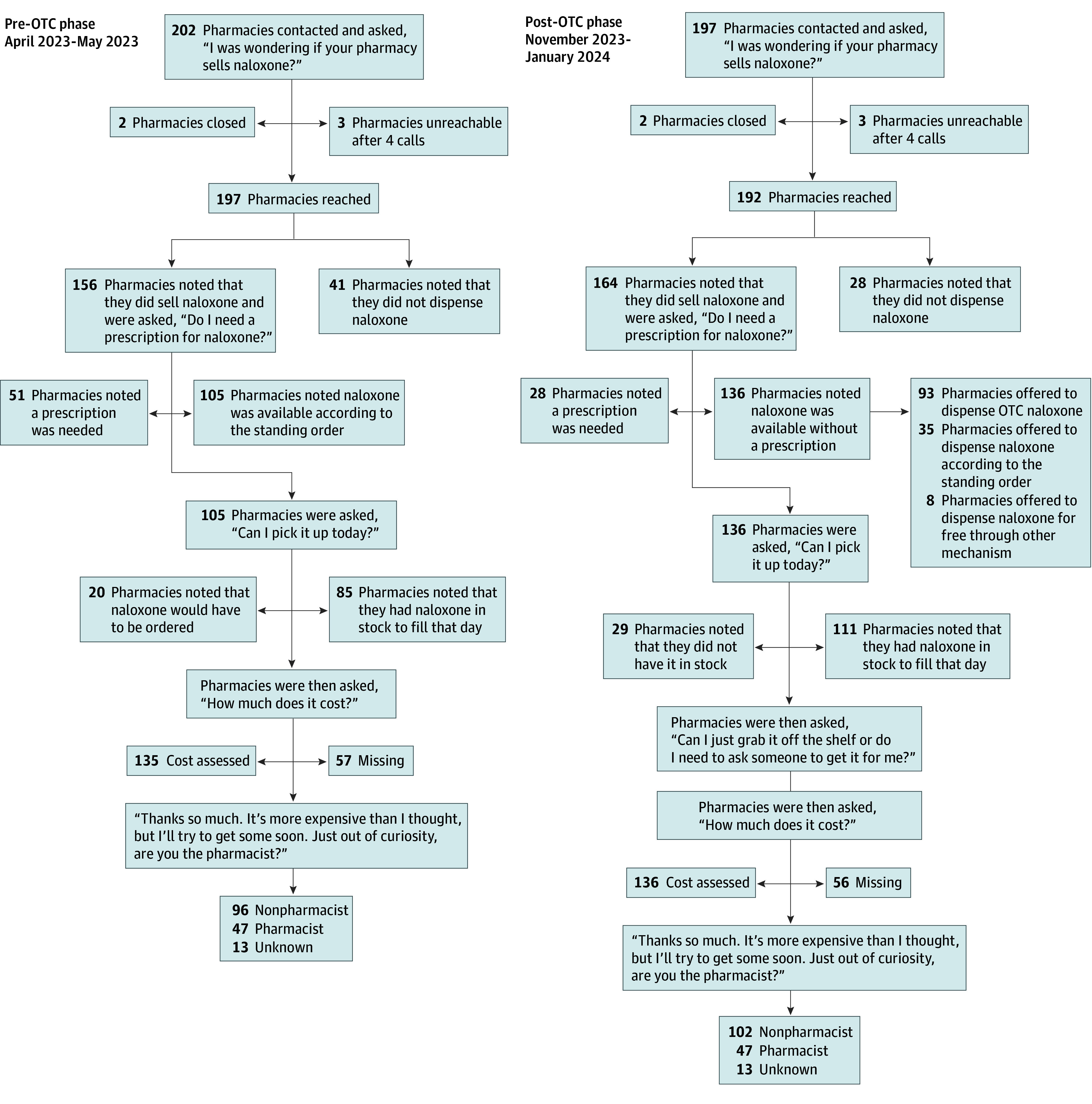
Secret Shopper Outline and Script OTC indicates over the counter.

In the pre-OTC phase, all secret shoppers enacted a standardized script that was based on the previous 2019 North Carolina secret shopper study script.^12^ In the post-OTC phase, the script was slightly modified based on feedback from an advisory panel of community experts to include questions specific to increasing understanding of OTC naloxone implementation, including a question regarding where in the pharmacy naloxone is located (Figure 1).

Prior to both data collection phases, shoppers met as a group to practice realistically enacting the script. The secret shoppers then met with the study coordinator to conduct practice calls with pharmacies not included in the sample and discuss procedures for coding interactions with pharmacy staff. Practice calls were conducted until the group achieved 100% consensus on observation guide ratings. During data collection, one of us (G.T.M.) met with secret shoppers to observe calls and provide feedback to ensure calls were being conducted with fidelity.

If shoppers were unable to reach pharmacy staff after 4 calls on different days and at various times, shoppers documented the pharmacy as unreachable, and these pharmacies were not included in the analysis. Successful calls were defined as those in which the call was answered, and pharmacy staff responded to the shopper’s questions.

### Measures

All data were recorded using an online observation guide managed in REDCap (Vanderbilt University).^31,32^ During each call, shoppers recorded if the pharmacy sold naloxone (yes or no) and if the pharmacy staff stated a prescription was needed to buy naloxone (yes or no). Shoppers documented if naloxone was available to pick up that day (yes or no). Shoppers also documented the quoted out-of-pocket cost of naloxone nasal spray. During calls in the post-OTC phase, shoppers also documented how the pharmacy offered to dispense naloxone (OTC, with a physician’s prescription, according to the statewide standing order, or other), as well as where in the pharmacy naloxone was located (behind the register, on the shelf, or other).

Data on pharmacy characteristics, including included pharmacy type (independent, chain, and health department) and pharmacy county, were derived from the NCBOP list. Pharmacies were geocoded using ArcGIS Online services (Esri) based on their physical address. Pharmacy urbanicity was measured based on the pharmacy’s zip code via rural-urban commuting area (RUCA) codes. RUCA code 1 was designated as urban, RUCA codes 2 and 3 were designated as suburban, and RUCA codes 4 to 10 were designated as rural.^33^ Uniform RUCA codes are a categorical standardized measure of urbanicity created by the Economic Research Service of the US Department of Agriculture.^33^

### Statistical Analysis

All analyses were conducted using SPSS version 28 (IBM). Descriptive statistics were calculated to describe pharmacy characteristics and naloxone availability. The McNemar test was used to compare naloxone availability in the pre-OTC and post-OTC phases. χ^2^ Tests were used to determine whether the proportion of pharmacies offering same-day naloxone differed by pharmacy type or by urbanicity. A paired samples *t* test was calculated to compare costs before and after OTC availability. A 1-way analysis of variance was run to determine if cost differed based on how naloxone was offered in the post-OTC phase (OTC, standing order, or physician’s prescription). Health department pharmacies were not included in the cost analysis due to the small sample size and differing dispensing mechanisms. Statistical significance was set at *P* < .05, and all *P* values were 2-tailed.

For each pharmacy, change in naloxone pricing was calculated by subtracting the quoted out-of-pocket cost in the post-OTC period from the pre-OTC period. Change in pricing was mapped using the geocoded pharmacy locations in R version 4.3.2 (The R Foundation).

## Results

### Sample Characteristics

A total of 202 pharmacies were selected for sampling. During the pre-OTC phase, 5 pharmacies were closed or unreachable. During the post-OTC phase, 5 additional pharmacies were closed or unreachable, yielding a final sample of 192 pharmacies for analysis, including 8 health department pharmacies, 97 chain pharmacies, and 87 independent pharmacies. In terms of urbanicity, 94 pharmacies were urban, 21 were suburban, and 77 were rural. Most pharmacies were reached on the first call in both the pre-OTC phase (187 [97.4%]) and post-OTC phase (185 [96.3%]).

### Naloxone Availability

There was a statistically significant increase in pharmacies’ willingness to dispense naloxone from the pre-OTC phase (152 [79.2%]) to the post-OTC phase (164 [85.4%]; *P* = .04) (Table 1). A total of 102 pharmacies (53.1%) were willing to dispense naloxone without a clinician-issued prescription during the pre-OTC phase as opposed to 136 pharmacies (70.8%) in the post-OTC phase (*P* = .003).

**Table 1.** Naloxone Availability at North Carolina Pharmacies Prior to and After It Became Available as an Over-the-Counter (OTC) Product

Measure	Phase, No. (%)^a^		*P* value
	Pre-OTC (n = 192)	Post-OTC (n = 192)	
Pharmacy willing to dispense naloxone	152 (79.2)	164 (85.4)	.04
Pharmacy will only dispense with clinician-issued prescription	50 (32.9)	28 (14.6)	.003
Pharmacy will dispense via statewide standing order	94 (49)	35 (18.2)	NA
Pharmacy has OTC product available	NA	93 (48.4)	
Health department dispensing	8 (4.1)	8 (4.1)	
Available the same day without clinician’s prescription	81 (42.2)	111 (57.8)	<.001

Abbreviation: NA, not applicable.

^a^ The pre-OTC phase was conducted from March to April 2023, and the post-OTC phase was conducted from November 2023 to January 2024.

Of the 93 pharmacies that were willing to dispense OTC naloxone during the post-OTC phase, 60 (65%) communicated that the OTC product was located behind the counter rather than on the shelf. Pharmacy willingness to dispense naloxone without a prescription was more common in chain pharmacies than independent pharmacies during both the pre-OTC phase (60 of 97 [62%] vs 34 of 87 [39%]; *P* = .003) and post-OTC phase (85 of 97 [88%] vs 44 of 87 [51%]; *P* < .001).

### Same-Day Availability of Naloxone

During the pre-OTC phase, only 81 pharmacies (42.2%) had naloxone in stock and were willing to dispense naloxone without a clinician-issued prescription compared with 111 (57.8%) in the post-OTC phase (*P* < .001). In the post-OTC phase, 11 pharmacies (11.8%) were willing to dispense OTC naloxone but needed to order the OTC formulation prior to dispensing.

Chain pharmacies had higher same-day naloxone availability without a clinician-issued prescription compared with independent pharmacies in the pre-OTC phase (50 of 97 [52%] vs 24 of 87 [27%]; *P* = .002) and post-OTC phase (77 of 97 [80%] vs 26 of 87 [30%]; *P* < .001) (Table 2). Same-day naloxone availability was not statistically different by pharmacy urbanicity in either the pre-OTC phase or the post-OTC phase.

**Table 2.** Differences in Naloxone Availability at Pharmacies by Pharmacy Type and Urbanicity in North Carolina Before and After Over-the-Counter (OTC) Naloxone Became Available

Measure	Pre-OTC phase, No./total No. (%)	*P* value	Post-OTC phase, No./total No. (%)	*P* value
Willing to dispense without a clinician-issued prescription	102 (53.1)	NA	136 (70.8)	.003
Pharmacy type				
Independent pharmacies	34/87 (39)	.10	44/87 (50.6)	<.001
Chain pharmacies	60/97 (61.9)		85/97 (87.6)	
Urbanicity				
Urban pharmacies	49/94 (52.1)	.97	71/94 (75.5)	.43
Suburban pharmacies	12/21 (57.1)		14/21 (66.7)	
Rural pharmacies	41/77 (53.2)		51/77 (66.2)	
Available the same day without a clinician-issued prescription	81 (42.2)	NA	111 (57.8)	<.001
Pharmacy type				
Independent pharmacies	24/87 (27.3)	<.001	26/87 (29.5)	<.001
Chain pharmacies	50/97 (52.1)		77/97 (80.2)	
Urbanicity				
Urban pharmacies	39/94 (41.5)	.87	60/94 (63.8)	.15
Suburban pharmacies	8/21 (38.1)		11/21 (52.4)	
Rural pharmacies	34/77 (44.2)		38/77 (49.4)	

Abbreviation: NA, not applicable.

### Quoted Out-of-Pocket Cost

The mean (SD; median) quoted out-of-pocket cost of naloxone nasal spray was lower in the post-OTC phase, decreasing from $90.43 ($42.5; $90) in the pre-OTC phase to $62.94 ($40.9; $45) in the post-OTC phase (*P* < .001) (Table 3). In the pre-OTC phase, mean (SD; median) naloxone cost was significantly higher at independent pharmacies ($109.47 [$37.9; $100]) compared with chain pharmacies ($86.40 [$35.7; $80]) (*P* < .001). Similarly, in the post-OTC phase, mean (SD; median) naloxone cost was higher at independent pharmacies ($77.59 [$38.4; $72]) compared with chain pharmacies ($57.74 [$35.9; $44.99]) (*P* = .004). Mean (SD) nonprescription naloxone costs were higher at independent pharmacies ($73.41 [$30.3]) compared with chain pharmacies ($50.64 [$18.4]) (*P* = .002).

**Table 3.** Quoted Out-of-Pocket Cost Differences Before and After Over-the-Counter (OTC) Naloxone Became Available by Dispensing Type, Pharmacy Type, and Urbanicity

Measure	Pre-OTC phase, mean (SD), $	*P* value	Post-OTC phase, mean (SD), $	*P* value
Cost	90.93 (42.6)	NA	62.67 (41.0)	<.001
Cost for naloxone dispensed by clinician’s prescription	100.79 (33.7)	.09	97.37 (67.9)	<.001
Cost for naloxone dispensed via statewide standing order	87.05 (45.8)		78.16 (42.5)	
Nonprescription OTC cost	NA	NA	56.85 (24.3)	
Pharmacy type				
Independent pharmacy	109.47 (37.9)	<.001	77.59 (38.4)	.004
Chain pharmacy	86.40 (35.7)		57.74 (35.9)	
Nonprescription OTC naloxone cost by pharmacy type				
Chain pharmacy	NA	NA	50.64 (18.4)	.002
Independent pharmacy	NA		73.41 (30.31)	
Prescription naloxone (standing order and clinician prescription) cost by pharmacy type				
Chain pharmacy	NA	NA	85.34 (65.43)	.88
Independent pharmacy	NA		82.45 (41.02)	
Cost by urbanicity^a^				
Urban	83.93 (45.8)	.15	53.58 (29.0)	.003
Suburban	93.34 (93.3)		88.67 (66.8)	
Rural	99.21 (99.2)		65.43 (35.0)	

Abbreviation: NA, not applicable.

^a^ Urbanicity was determined by rural-urban commuting area codes, with urban defined as code 1, suburban as codes 2 and 3, and rural as codes 4 to 10.^33^

**Figure 2.**
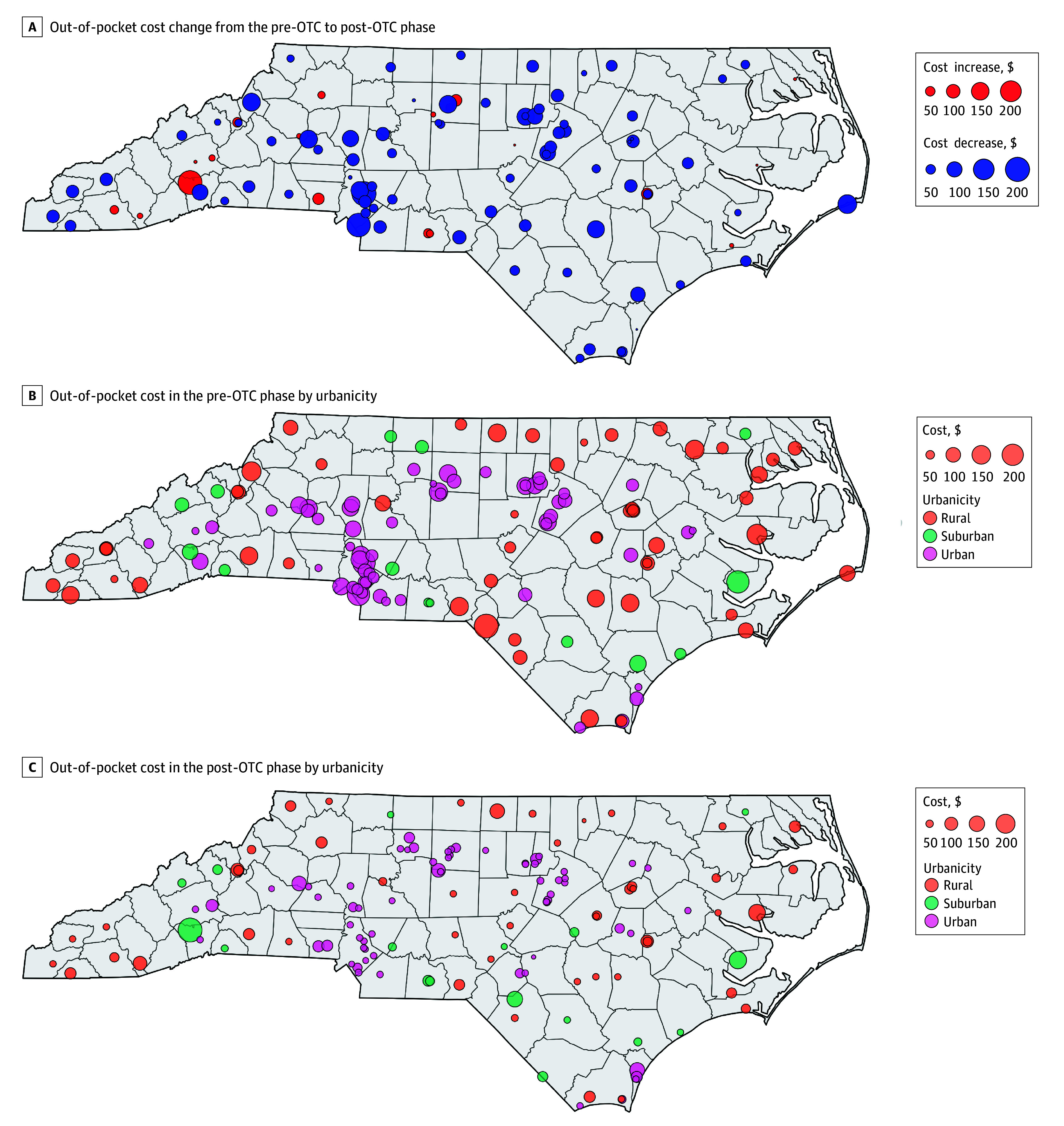
Maps of Naloxone Availability Before and After Introduction of Over-the-Counter (OTC) Naloxone to the Market and Mapped Changes in Quoted Out-of-Pocket Cost Urbanicity was determined by rural-urban commuting area codes, with urban defined as code 1, suburban as codes 2 and 3, and rural as codes 4 to 10.^33^

Mean (SD; median) quoted out-of-pocket costs in the pre-OTC phase were not significantly higher in rural areas ($99.21 [$39.5; $99.99]) compared with suburban areas ($93.34 [$38.3; $85.51]) and urban areas ($83.93 [$45.8; $85]) (*P* = .15). However, in the post-OTC phase, suburban pharmacies had higher mean (SD; median) costs ($88.67 [$66.8; $63.99]) than rural ($65.43 [$35.1; $50]) and urban ($53.58 [$29.0; $45]) pharmacies (*P* = .003) (Figure 2).

## Discussion

This study confirms that pharmacy willingness to dispense naloxone without a clinician-issued prescription increased after OTC naloxone became available in North Carolina pharmacies. This study also found that cost was significantly reduced following OTC naloxone availability. Similar to previous research, we found elevated out-of-pocket costs for naloxone nasal spray at independent pharmacies compared with chain pharmacies during both phases of the study.^4,5,9,10,15,24,25,26^ Lastly, although naloxone availability did not differ significantly by urbanicity, rural and suburban pharmacies charged significantly more for naloxone in the post-OTC phase.

Access to naloxone without a clinician-issued prescription decreased from 67.1% of North Carolina pharmacies surveyed in 2019 to only 53.1% in 2023, but after the introduction of OTC naloxone, approximately 70.8% of North Carolina pharmacies were willing to dispense without a prescription.^12,15^ Although this is a significant increase in willingness to dispense compared with studies conducted prior to OTC naloxone availability, only 58% of pharmacies in the post-OTC sample had naloxone available for same-day pick-up.^13^ These findings suggest that OTC naloxone availability in the marketplace is not sufficient to ensure convenient pharmacy-based access to naloxone. Naloxone dispensing barriers identified previously may still exist, such as a perceived lack of time to educate customers, difficulty stocking naloxone, inadequate pharmacist training, and stigma surrounding opioid use disorder.^6,7,8,9,10,11,20,21^ Additional research is needed to evaluate the specific barriers to same-day OTC naloxone dispensing and opportunities to reduce these barriers.

At both time points, chain pharmacies were more likely than their independent counterparts to dispense naloxone without a clinician-issued prescription and charge less for naloxone nasal spray. These findings are like secret shopper studies conducted before OTC naloxone was available, which found limited naloxone availability in independent pharmacies compared with chain pharmacies.^4,12,13,15,17,31,32^ Official naloxone stocking policies and procedures that oftentimes exist in chain pharmacies, such as automatic ordering for medications, may be lacking in independent pharmacies, which may drive this discrepancy in availability.^34,35^ Educational programming that emphasizes the importance of naloxone dispensing should be targeted for independent pharmacists in an attempt to increase access.

The quoted out-of-pocket cost of naloxone significantly decreased after OTC naloxone became available, with a mean decrease in price of almost $30, even with the inclusion of prescription and nonprescription drug prices. Interestingly, the mean quoted out-of-pocket cost for OTC naloxone was still higher than the suggested manufacturer’s retail price of $44.99 for a 2-pack Narcan nasal spray^29^ and was higher at independent pharmacies than chain pharmacies. Pharmacies can set their own prices for OTC medications. It is possible that given this freedom, independent pharmacies chose to increase the cost of this nonprescription product above the suggested retail price, which could explain why independent pharmacies are charging approximately $20 more on average compared with chain pharmacies. Alternatively, calling pharmacies only 2 months after OTC naloxone had become available may have provided limited time for pharmacies to sell their existing stock of prescription naloxone and transition to selling OTC naloxone, which may have affected price. It is possible that more pharmacies will carry OTC naloxone in the future, which could further lower quoted costs. Additional research should be conducted to evaluate independent and chain pharmacy nonprescription product pricing strategies and how these strategies impact patient access and affordability of naloxone.

Naloxone access via harm reduction organizations is often limited in rural areas, further increasing the need for pharmacy-based naloxone access.^36,37^ Unlike previous secret shopper studies, we did not find differences in naloxone availability by urbanicity.^9,13,38^ However, the cost of naloxone nasal spray was higher in rural and suburban areas. This is of particular importance in North Carolina, where the drug overdose death rate is higher in rural counties.^39^ Increased naloxone costs in rural areas can result in patient inability to afford naloxone and contribute to increased overdose death rates.

### Limitations

This study has several limitations. Phone-based data collection could have yielded different results than in-person data collection. The restriction to North Carolina pharmacies could limit the generalizability of findings; however, previous studies have shown that North Carolina pharmacy access and pricing are similar to that of other states.^4,5,9,10,15,25,26^ Price analyses would likely be different if insurance copayments were assessed. However, these results capture the expected cost for those most vulnerable to opioid overdose, people who use drugs, who often do not have health insurance. Future studies should evaluate how naloxone costs have changed for insured individuals. The use of RUCA codes for the designation of urbanicity may skew cost and availability analyses, as these designations may not fully encompass self-reported urbanicity.^40,41^ The pre-OTC call may have been perceived by pharmacy staff as an increased demand for naloxone, which could have led to increases in availability during the post-OTC phase. Future secret shopper studies should evaluate whether the trends in increased availability and decreased cost continue.

## Conclusions

The Food and Drug Administration’s approval of OTC naloxone nasal spray may have contributed to an increase in pharmacy-based availability of naloxone and a reduction of its cost at North Carolina pharmacies. This study found elevated out-of-pocket costs for naloxone at independent pharmacies compared with chain pharmacies and in rural and suburban pharmacies compared with urban pharmacies. Overall, future work should evaluate OTC naloxone pricing strategies at pharmacies and evaluate methods to increase its same-day availability.

## Notes

Supplement. Data Sharing Statement
