# Supplementary material for: Naloxone Availability and Cost After Transition to an Over-the-Counter Product
Source: JAMA Health Forum. 2024 Jul 26;5(7):e241920. doi: 10.1001/jamahealthforum.2024.1920 (PMC11282446; doi:10.1001/jamahealthforum.2024.1920)
Supplement: Supplement. — Data Sharing Statement [file jamahealthforum-e241920-s001.pdf]

Data Sharing Statement

Marley. Naloxone Availability and Cost After Transition to an Over-the-Counter Product. *JAMA Health Forum*. Published July 26, 2024. doi:10.1001/jamahealthforum.2024.1920

Data
Data available: No
